# Rapid adaptive evolution of oxylipin-based chemical defense against algicidal bacteria in a bloom-forming diatom

**DOI:** 10.1093/ismejo/wrag111

**Published:** 2026-05-08

**Authors:** Muhaiminatul Azizah, Janine F M Otto, Nico Ueberschaar, Markus Werner, Oliver Werz, Georg Pohnert

**Affiliations:** Bioorganic Analytics, Institute for Inorganic and Analytical Chemistry, Friedrich Schiller University, Lessingstrasse 8, D-07743 Jena, Germany; Bioorganic Analytics, Institute for Inorganic and Analytical Chemistry, Friedrich Schiller University, Lessingstrasse 8, D-07743 Jena, Germany; Mass Spectrometry Platform, Friedrich Schiller University Jena, Humboldtstr. 8, 07743 Jena, Germany; Department of Pharmaceutical/Medicinal Chemistry, Institute of Pharmacy, Friedrich Schiller University Jena, 07743 Jena, Germany; Department of Pharmaceutical/Medicinal Chemistry, Institute of Pharmacy, Friedrich Schiller University Jena, 07743 Jena, Germany; Bioorganic Analytics, Institute for Inorganic and Analytical Chemistry, Friedrich Schiller University, Lessingstrasse 8, D-07743 Jena, Germany

**Keywords:** adaptive laboratory evolution, chemical defense, diatoms, algicidal bacteria, metabolomics, oxylipins, plankton

## Abstract

Phytoplankton, the photosynthetic microalgae driving nearly half of Earth’s primary production, are the foundation of marine food webs and central to climate regulation. *Skeletonema marinoi* is a globally distributed and often dominant diatom species in marine phytoplankton communities. It inhabits a dynamic and frequently hostile microbial environment in which interactions with bacteria can negatively affect its survival. *Skeletonema marinoi* is susceptible to the algicidal bacterium *Kordia algicida* that can lyse the algal cells and even terminate entire blooms. The extent to which resistance against such a lysis can evolve in diatoms exposed to biotic stress by algicidal bacteria remains unknown. Using adaptive laboratory evolution, we investigated how *S. marinoi* adapts to toxins produced by *K. algicida. Skeletonema marinoi* evolved resistance already after 11 growth cycles under sublethal exposure to algicides. This was accompanied by changes in DNA methylation. Untargeted comparative metabolomics of the original and the evolved population revealed the up-regulation of the oxylipins 5-hydroxyeicosapentaenoic acid, prostaglandin E_2_, and 17-hydroxydocosahexaenoic acid. These oxylipins significantly inhibited the growth of *K. algicida*, indicating their role in chemical defense. The metabolic plasticity of diatoms and the rapid evolution observed after exposure to bacteria open new perspectives on our understanding of diatom bloom dynamics in nature.

## Introduction

Plankton communities comprise highly complex species assemblages fueling marine and freshwater food webs [1]. Photosynthetic activity in the plankton is dominated by phytoplankton that contributes nearly 50% to the global carbon fixation [2, 3]. The diatom *Skeletonema marinoi* is a ubiquitous component of marine phytoplankton with a global distribution in temperate coastal waters. It frequently forms large-scale blooms, e.g. in the Baltic Sea, East China Sea, and Mediterranean Sea, where it plays a key role in ecosystem productivity and nutrient cycling [4, 5]. *Skeletonema marinoi* exists in a dynamic and often hostile microbial environment, where interactions with bacteria can profoundly influence its survival, physiology, and bloom dynamics [5]. *Kordia algicida* is a well-studied algicidal bacterium that was isolated from a collapsing *S. marinoi* bloom [6]. The bacterium lyses *S. marinoi* and several other microalgal species by means of proteases and other, yet unknown, chemical mediators. The heterotrophic bacterium then feeds on the released resources. Because several other microalgae in the plankton are resistant, this selective action results in a bacteria-mediated change in the microbial community [6–9]. Resistant phytoplankton species can escape lysis and grow in the presence of *K. algicida*, providing a selective advantage over susceptible species [10, 11]. Defense strategies to survive bacterial antagonism are often based on the production of chemical mediators that inhibit bacterial proliferation or activity [12, 13]. For example, the diatom *Chaetoceros didymus* defends itself by producing oxylipins that inhibit the growth of *K. algicida* as well as proteases that neutralize algicidal proteases from the bacterium [10, 14].

A key goal in plankton ecology is to understand the complex community composition and succession, which provides mechanistic insights into ocean functioning. We do not know how different individual cells of *S. marinoi* within a bloom might adapt to the presence of harmful bacteria and if survivors can guarantee the observed microbial diversity in seawater. Over time, exposure to bacterial antagonists like *K. algicida* might impose selective pressure, potentially driving the adaptive evolution of resistance traits in diatom populations. On evolutionary timescales, these adaptations may lead to stable inheritance of resistance traits, enabling diatoms to withstand algicidal attack. Previous research has, however, only focused on the algicidal mechanisms from the bacterial perspective and on the physiological response of resistant diatoms [10, 15]. There is, however, no study that investigates the possible evolved resistance of a diatom during long-term exposure to antagonists, which would teach us about fundamental principles of plankton succession. We therefore set out to conduct an adaptive laboratory evolution experiment to determine the plasticity and evolution of chemical defense mechanisms of *S. marinoi*. We reasoned that understanding of how the alga adaptively evolves following repeated exposures to *K. algicida* is a key to unraveling the co-evolutionary dynamics between phytoplankton and bacteria.

Adaptive laboratory evolution (ALE) is an experimental strategy used mainly in biotechnology to improve the performance of microbial cultures. In this approach, microbial populations are repeatedly exposed to sublethal abiotic or biotic stress conditions. Cells that survive these challenges are selected to initiate the next growth cycle. Over many cycles, this process favors the accumulation of beneficial genetic, epigenetic, and physiological changes [16]. Initially sensitive strains can gradually develop increased resistance or strains can be selected for enhanced productivity. In algae, evolved responses to environmental factors, such as acidification, warming, elevated *p*CO_2_, or salinity, have been addressed through adaptive evolution, mainly with the aim to increase productivity in aquaculture [17–20]. We reasoned that ALE could also serve as an approach to investigate algal responses to biotic challenges and their potential ecological consequences in planktonic systems. Therefore, this study aims to investigate the adaptive evolution of resistant traits in *S. marinoi* exposed to sublethal lytic factors of the algicidal bacterium *K. algicida*. We focus on the identification and functional characterization of chemical mediator**s** involved in this process by using comparative metabolomics.

## Materials and methods

### Purchased materials

Commercially available standards 5-(*R*-*S*)-hydroxyeicosapentaenoic acid (HEPE), 15-(*R*-*S*)-HEPE, 15-(*S*)-HEPE, 18-(*R*-*S*)-HEPE, 17-(*R*-*S*)-hydroxydocosahexaenoic acid (HDHA), and prostaglandin E_2_ (PGE_2_) were obtained from Cayman Chemical. Acetonitrile (Th Geyer GmbH), ammonium acetate (LGC Promochem), ammonium formate (LGC Promochem), formic acid (Thermo Scientific), water (Th Geyer GmbH), and methanol (VWR Chemicals) used in this experiment were purchased in LC–MS grade. Water used for media preparation was purified using a Milli-Q system (Millipore). Internal standards for DNA methylome analysis were purchased from Toronto Research Chemicals Inc. (2′-deoxycytidine-^13^C_1_, ^15^N_2_ (C-^13^C_1_^15^N_2_), 2′-deoxy-5-methylcytidine-^13^C_1_, ^15^N_2_ (5mC-^13^C_1_^15^N_2_), and 2′-deoxy-*N*-6-methyladenosine-d^3^ (6mAd3)) and Cambridge Isotope Laboratories, Inc. (2′ deoxyadenosine-^15^N_5_ (A-^15^N_5_)).

### Phyto- and bacterioplankton strains and growth conditions

Phytoplankton *S. marinoi* (accession number RCC75) was purchased from the Roscoff Culture Collection (https://roscoff-culture-collection.org/) and cultivated in artificial seawater medium (ASW) [21], with a salinity of 35 PSU, at 13°C, 14:10 light–dark cycle with light intensity range 15–30 μmol/m^2^/s [15]. The bacterium *K. algicida* (accession number NBRC 1000336) was obtained from the Biological Resource Center, NITE (NBRC), and stored as a cryo stock containing 50% glycerol. The cultivation of *K. algicida* followed a published protocol [15]. Briefly, bacterial cultures for each experiment were initiated from cryo stock by streaking onto marine broth agar plates and incubating at 28°C for 2–3 days. Single colonies were then transferred to marine broth (Carl Roth) and grown at 28°C with 80–100-rpm shaking until the mid- to late exponential phase (OD_550_ of 0.2 corresponding to ~1.6 × 10^6^ cells ml^−1^) was reached [15].

To determine the growth of *S. marinoi, in vivo* chlorophyll (chl *a*) fluorescence (excitation wavelength of 430 nm and emission wavelength of 665 nm) was measured in technical triplicates on a Varioskan Flash plate reader (Thermo Fisher Scientific). To determine the growth of *K. algicida*, the absorbance (OD_550_) was measured using a spectrophotometer (Analytik Jena) and a Varioskan Flash plate reader (Thermo Fisher Scientific). For normalization of metabolomics experiments, phytoplankton cells were counted under a Leica DM 2000 light microscope in a Fuchs–Rosenthal counting chamber.

### Preliminary lysis study using cell-free spent medium

Bacterial cultures were incubated in marine broth media at 28°C to reach an OD_550_ of 0.8. Then, each 7.5-ml aliquot of bacterial cultures was diluted into (a) 300 ml of marine broth:ASW (1:20 *v/v*) and (b) 300 ml of marine broth:ASW (1:10 *v/v*). Then, the cultures were incubated at 28°C with a starting OD_550_ of 0.02 for 48 h. Bacterial cultures were centrifuged for 30 min at 2900 rpm at 15°C for 15 min. The supernatant was filtered through a PES membrane filter with a pore size of 0.2 μm under reduced pressure (500 mbar). The filtrate was used as a cell-free spent medium (CFSM) for the preliminary study. Both CFSM containing marine broth:ASW (1:20 *v/v*) and marine broth:ASW (1:10 *v/v*) were used for this study separately, with compositions of a *S. marinoi* culture with 1.5 × 10^5^ cells ml^−1^:CFSM (20:4, 20:6; 20:8, 20:10, 20:20 *v/v*). The chl *a* fluorescence was recorded from days 1 to 4 after inoculation. Cultures containing no CFSM were used as a control. The goal was to allow the phytoplankton cells to reach ~50% mortality after the addition of CFSM, ensuring that enough viable cells remain to be re-cultured in fresh ASW media for subsequent experiments. According to this preliminary study, we decided to use CFSM from bacterial cultures that were incubated in medium containing marine broth:ASW (1:20 *v/v*), with an incubation time of 1 day (Supporting Fig. 1). Marine broth:ASW (1:20 *v/v*) was active, in accordance with the reported high algicidal activity under nutrient starvation determined previously by dilution experiments of marine broth in seawater as *K. algicida* culture medium [15].

### Batch production of CFSM from the bacterium *K. algicida*

Bacterial cultures were incubated in marine broth medium at 28°C until OD_550_ 0.8. Then, 30-ml aliquots of bacteria were diluted into 1200 ml of medium containing marine broth:ASW (1:20 *v/v*) to reach a starting OD_550_ of ~0.02. Then, the culture was incubated at 28°C for 48 h to reach an OD_550_ of 0.2. Bacterial cultures were centrifuged for 30 min at 2900 rpm at 15°C. The supernatant was filtered through a 0.2-μm PES membrane under reduced pressure (500 mbar). The filtrate constitutes the CFSM for the ALE experiments.

### Adaptive laboratory evolution of phytoplankton

**Figure 1:**
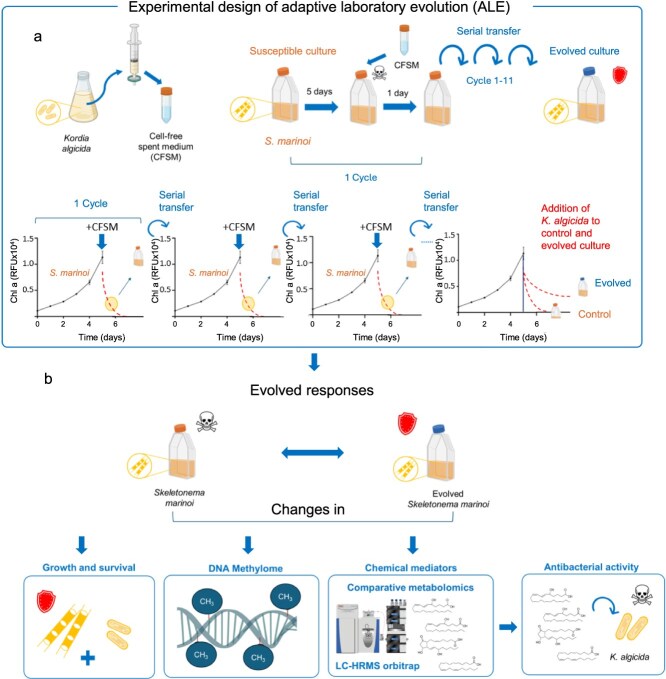
Experimental workflow of the adaptive laboratory evolution (ALE) experiment. (a) The cell-free spent medium (CFSM) was obtained from *K. algicida* cultures by sterile filtration. *Skeletonema marinoi* cultures were grown for 5 days to the mid-exponential phase and then treated with CFSM. Incubation for 24 h resulted in the lysis of approximately half of the algal cells. The surviving cells were transferred to fresh medium. This process was defined as 1 cycle. The cycle was repeated until the evolved cultures exhibited increased resistance to CFSM compared to the untreated *S. marinoi* controls. (b) The evolved response of *S. marinoi* was investigated in bioassays comparing survival toward CFSM in comparison to a control. Also, changes in the DNA methylation and the endometabolome in the evolved compared to the control strains were evaluated. The identified putative chemical mediators of phytoplankton resistance were evaluated in bioassays with *K. algicida*.

Phytoplankton *S. marinoi* was cultivated in 20 ml of ASW with a starting Chl *a* of 0.1 RFU × 10^4^ for 5–6 days until it reached the exponential phase (Chl *a* ~1.0 RFU × 10^4^). Phytoplankton cultures were then amended with 8 ml of CFSM of *K. algicida* prepared as described above and incubated for 24 h. The procedure triggered ~50% mortality of the algae, ensuring that enough viable cells remain to be re-cultured. Of these cultures, 10 ml was centrifuged at 2600 rpm, the supernatant was discarded, and the remaining cells were washed with fresh ASW. Washing and centrifugation were repeated twice to remove residual CFSM and then added to 10 ml of fresh ASW. Around 2 ml of cultures was then transferred into 20 ml of fresh ASW medium (with a starting Chl *a* of 0.1 RFU × 10^4^) to start the next cycle. The process involving the cultivation of *S. marinoi* until reaching the exponential phase, incubation with CFSM, and collection of the surviving cells to start a new culture is defined as 1 cycle. The cycle was repeated until the evolved cultures developed resistance toward the algicidal bacterium compared to susceptible *S. marinoi* (Fig. 1).

### Extraction and sample preparation

For comparative metabolomics, evolved and control phytoplankton cultures (controls were not exposed to CFSM but otherwise grown and diluted in identical cycles since the beginning of the ALE) were harvested in the mid-exponential growth phase with a density of 1.5 × 10^5^ cells ml^−1^ (determined in independent preliminary experiments recording the cell count and chlorophyll *a* content) by filtration on 25-mm Whatman GF/C grade microfiber filters [22]. For each biological replicate, 40 ml was filtered under reduced pressure (500 mbar). The filter was immediately transferred into 4-ml glass vials containing 500 μl of methanol, and another 500 μl of methanol was added directly to the filter. The vials were closed with a Teflon-lined cap and vortexed for 30 s. The methanol phase was transferred to new tubes by pipetting and centrifuged for 20 min at 16 100×*g*. Organic phases were transferred into new glass vials and dried under a stream of nitrogen.

### Untargeted comparative metabolomics using LC-HRMS

The dried extracts were taken up with 90 μl of methanol, and 11 μl of each sample was transferred into a quality control (QC) mix sample. One microliter of the respective samples or the QCs was injected into an ultra-high-pressure liquid chromatography–high-resolution mass spectrometer (UHPLC–HRMS) consisting of a UHPLC Vanquish equipped with an Accucore C-18 column (100 × 2.1 mm, 2.6 μm) coupled to an Orbitrap Exploris 480 mass spectrometer (Thermo Fisher Scientific). The chromatographic separation was performed according to a modified previously published method [23]. Separation was performed using a 12-min gradient, starting with 100% of the aqueous phase (2% acetonitrile, 0.1% formic acid in water) and increasing with the acetonitrile phase (0.1% formic acid in acetonitrile) within 8 min until reaching 100%. This was held for 3 min before switching back to 100% of the water phase and equilibration for 1 min. The flow rate was set at 0.4 ml min^−1^. Electrospray ionization was performed in the negative polarity mode at 2.5 kV. Sheath gas was 50, auxiliary gas was 10, and sweep gas was 1 (arbitrary units). The default charge state was 1 and the expected peak width was 6 s. Mass spectrometry was conducted with a scan range of *m/z* 80 to 1200 at a peak resolution of 90 000. Automatic gain control (AGC) target was set to standard. MS/MS spectra were acquired by data-dependent high-resolution tandem mass spectrometry (ddMS^2^ Top 5) within an isolation window of *m/z* 0.4 at a peak resolution of 15 000 (HCD collision energy 30, 60, 90). 5-HEPE, 17-HDHA, PGE_2_, EPA, arachidonic acid, and DHA were identified by comparing retention time (RT), MS, and MS/MS spectra of compounds in the algal extracts to commercial standards. The raw dataset was uploaded to the GNPS public repository, under accession number MSV000099304.

### Data processing and analysis of significantly regulated metabolites

UPLC–HRMS raw data were recorded using the Xcalibur software (Thermo Fisher Scientific). Raw data were imported into the Compound Discoverer software 3.3.3.200 (Thermo Fisher Scientific) for peak picking, deconvolution, identification of the metabolites, and multivariate statistical analysis. The parameters are according to previously published methods [24, 25]. The mass tolerance for MS identification was 5 ppm, the minimum peak intensity was set at 1 × 10^5^, the peak rating threshold was set to 2, and the RT tolerance was 0.2 min. Precursor mass tolerance was set at 0.025 Da and the fragments’ mass tolerance was 5 ppm, with a chromatographic S/N threshold of 1.5. Structure prediction/annotation was performed using MassList search, mzCloud search (cosine algorithm was set for identity search in DDA), ChemSpider search, mzVault, and Predicted Composition. For all tools used for spectrum similarity searches, the mass tolerance was set to 5 ppm. The mzCloud search was used to compare MS^2^ spectra from ID files with an extensively curated, high-quality mass spectral fragmentation library, and mzLogic combines the extensive fragmentation information available within the mzCloud online advanced mass spectral library with the information in several online structural databases [24]. The parameter “scale areas” was set for cell count normalization. Features that were also identified in blank samples were removed. Pooled QC samples were used to compensate for time-dependent batch effects during analysis [26]. For post-processing nodes, differential and descriptive analyses were set for statistical analysis. Differential analysis was set with the *P*-value of the per group ratio calculated by two-tailed Student’s *t-*test, log-10 areas for *P*-value estimation, and *P*-value adjusting using Benjamini–Hochberg correction for the false-discovery rate. Log_10_ transform value was ticked on the “differential analysis” parameter. Volcano plots were utilized to display the univariate analysis results for each LMWC with adjusted *P* <0.05 and a fold-change >2. Supporting Table 1 lists the injection sequence of the samples, cell density, and normalization factors. Following this analysis, univariate statistical tests (principal component analysis, PCA) were completed using MetaboAnalyst 6.0 [27].

The identification of selected features was based on the MS/MS spectra (Supporting Table 2) that were compared by spectral similarity search with those found in mzCloud and with analytical standards. Assignment of putative identities of selected ions was performed through a database search of selected MS^2^ spectra in mzCloud. If possible, the actual identity was confirmed by comparison of retention time and MS^2^ spectra of an analytical standard.

### Oxylipin profiling

Samples (see section untargeted comparative metabolomics) were stored at −20°C. Then, 10 μl of each sample was injected and oxylipins were analyzed using an UHPLC–MS/MS system combining an Acquity UPLC (Waters, Milford, MA) and a QTRAP 5500 mass spectrometer (ABSciex, Darmstadt, Germany) as previously described [28]. Oxylipins were separated on an ACQUITY UHPLC BEH C18 column (1.7 μm, 2.1 mm × 100 mm; Waters, Eschborn, Germany) at 50°C with a flow rate of 0.3 ml min^−1^ and a mobile phase consisting of methanol–water–acetic acid (starting at 42:58:0.01, v/v/v) that was ramped to 86:14:0.01 over 12.5 min and then to 98:2:0.01 for 3 min. The QTRAP 5500 was operated in negative ionization mode using scheduled Multiple Reaction Monitoring for oxylipin quantification coupled with information-dependent acquisition and Enhanced Product Ion scans for confirmation of the chemical constitution of oxylipins (MS^2^ spectra with diagnostic ions after fragmentation). The retention time and at least six diagnostic ions for each oxylipin were confirmed by means of external standards (Cayman Chemical/Biomol GmbH, Hamburg, Germany), which were included in a QC sample measured in parallel to the unknown samples. Quantification was performed using calibration curves for each oxylipin normalized to the corresponding physicochemical-related internal isotope labeled standard (IS).

### Chiral analysis of 5-HEPE

Chiral UHPLC–HRMS was carried out using a Thermo (Bremen, Germany) UltiMate HPG-3400 RS binary pump and WPS-3000 auto sampler which was set to 10°C and which was equipped with a 25-μl injection syringe and a 100-μl sample loop. The Daicel Chiralpak AD-RH (150 × 4.6 mm; 5 μm) column was kept at 25°C within the column compartment TCC-3200. Elution was done at a constant flow rate of 0.4 ml/min with a gradient of water with 2% acetonitrile and 0.1% formic acid (eluent A) and pure acetonitrile (eluent B). The gradient started with 50% B for 0.2 min, ramped to 100% B at 8 min, held at 100% B until 13 min, and re-equilibrated at 50% B until 15 min.

Mass spectra were recorded with a Thermo QExactive plus orbitrap mass spectrometer coupled to a heated electrospray source. Column flow was switched at 0.5 min from waste to the MS and at 14 min again back to the waste, to prevent source contamination. For monitoring, two full-scan modes were selected with the following parameters. Polarity: negative; scan range: 200 to 1500 *m/z*; resolution: 70 000; AGC target: 3 × 10^6^; maximum IT: 200 ms. General settings: sheath gas flow rate: 60; auxiliary gas flow rate: 20; sweep gas flow rate: 5; spray voltage: 2.5 kV; capillary temperature: 320°C; S-lens RF level: 90; auxiliary gas heater temperature: 400°C; acquisition time frame: 0.5–11.5 min. MS^2^ spectra settings: parallel reaction monitoring, fixed first mass: 50, resolution: 17 500; AGC target: 2 × 10^5^; maximum IT: 100 ms; preferred charge state = 1. A collision energy with 15 NCE was used at an isolation window of 0.4 *m/z*.

### Bioassays

Bioassays are based on a previously reported method [10]. Briefly, in 96-well plates, 50-μl aliquots of a *K. algicida* culture were added to 15-HEPE, 5-HEPE, 17-HDHA, PGE2, and arachidonic acid, respectively, in 150 μl marine broth medium to result in a final concentration of 1 μg/ml, each in four biological replicates. Plates were shaken at 130 rpm and then sealed with Parafilm. After 24 h at 28°C, the OD_550_ was measured using a Varioskan Flash plate reader (Thermo Fisher Scientific). *Kordia algicida* growth in the treatments is assessed relative to that in the control.

### DNA methylome analysis

DNA methylome analysis was carried out following a modified published protocol [29]. For DNA extraction, 25 ml of each of the three susceptible and resistant cultures were centrifuged at 4000 rpm for 15 min at 15°C. After discarding the supernatant, the algal cells were freeze-dried in an Alpha 1-2 LDplus (Christ; Sigma Laborzentrifugen GmbH, Osterode, Germany) overnight and stored at −20°C until DNA extraction. Directly before DNA extraction, three 1.5-mm metal beads were added to the algal cells, and they were treated twice for 1 min at 30/s with the TissueLyser II (Qiagen, Germany) to disrupt the cells. Then, 4 μl of RNase A solution (100 mg/ml) was added during the cell lysis step. Genomic DNA was extracted using the DNeasy Plant Mini Kit (Qiagen, Germany) following the general protocol. The DNA was eluted with 100 μl DNase and RNase-free water (Th. Geyer, Germany). The DNA concentration was determined using a Qubit 3.0 fluorometer (Thermo Fisher Scientific, Bremen, Germany), and DNA was stored at −20°C until further steps.

For DNA hydrolysis, algal genomic DNA (50 ng) was aliquoted into micro-inserts (0.1 ml, 15 mm; VWR International GmbH, Darmstadt, Germany) in glass vials (1.5 ml, 8 mm; WICOM Germany GmbH, Heppenheim, Germany). The reaction mixture was prepared by adding 10 μl of an internal standard solution (final concentration: 1 μM A-^15^N_5_, 0.5 μM C-^13^C_1_^15^N_2_, 5 nM 5mC-^13^C_1_^15^N_2_, and 5 nM 6mAd^3^) and 10 μl of 8% HCl before adjusting to a total volume of 40 μl with deionized water (final acid concentration: 2%). Vials were sealed with a Teflon cap and incubated at 120°C for 3 h. After cooling to room temperature and freeze-drying, the resulting hydrolysates were redissolved in 40 μl of deionized water and analyzed using UHPLC–HRMS. The analysis followed a published procedure with the slight modifications indicated below [29]. For UHPLC separation, an UltiMate 3000 UHPLC system (Thermo Fisher Scientific) with a HPG-3400 rapid separation binary pump, TCC-3200 column department, and WPS 3000 autosampler was used. Then, 2 μl of the samples was injected using an autosampler at 10°C and equipped with a 25-μl injection syringe and 100-μl sample loop. A Phenomenex Synergi Fusion-RP (100 × 2 mm, 2.5 μm) column with the following gradient was used to separate the nucleobases: 100% A (20 mM ammonium formate, pH 3.8) for 0.2 min, linear gradient to 50% B (acetonitrile) in 4 min, column rinsing for 2 min with 100% B, and re-equilibration with 100% A for 1 min at a constant flow rate of 0.4 ml/min.

Mass spectra were recorded with a Thermo Scientific Q Exactive plus hybrid quadrupole-Orbitrap mass spectrometer (Thermo Fisher Scientific) equipped with a heated electrospray source. The analysis used the following parameters: scan range: 80–800 *m/z*, resolution: 35 000, AGC target: 5 × 10^5^; maximum injection time (IT): 64 ms. General settings: sheath gas flow rate: 60; auxiliary gas flow rate: 20; sweep gas flow rate: 6; spray voltage: 3.0 kV; capillary temperature: 360°C; S-lens radio frequency (RF) level: 50; auxiliary gas heater temperature: 400°Cni; acquisition time frame: 0.4–7 min. Methylated bases can be readily identified by their characteristic masses (+14 amu compared to the non-methylated forms) and retention times [29].

MS-Data analysis was performed using Thermo Scientific FreeStyle version 1.8SP2 and Xcalibur version 4.5.445.18 Quan Browser software (Thermo Fisher Scientific). The following settings were made for peak detection and quantification of the nucleobases: retention time window = 20 s; signal = XIC from full scan; peak detection algorithm = ICIS (smoothing = 9); peak detection mode = highest peak; mass tolerance = ±5.0 ppm. The following equation was used to calculate the relative amounts of methylated nucleobases in percent:


$$\frac{c\left(\mathrm{methylated}\ \mathrm{base}\right)}{c\left(\mathrm{methylated}\ \mathrm{base}\right)+c\left(\mathrm{unmodified}\ \mathrm{base}\right)}\times 100$$

The samples were measured in biological triplicates with three technical replicates each, resulting in nine data points per condition. All calibration curves used for absolute quantification of 5mC and 6mA are reported in Supporting Fig. 10.

## Results and discussion

### Adaptive laboratory evolution (ALE) study of *S. marinoi* under prolonged exposure to algicidal bacterial metabolites

In this study, serial culture dilution [19] was developed as an ALE tool in which a *S. marinoi* culture was repeatedly exposed to sublethal concentrations of CFSM from *K. algicida* (Fig. 1). We initially generated sufficient spent medium from bacterial cultures with algicidal activity [15] to conduct a prolonged evolutionary study. In preliminary experiments, we determined the amount of algicidal medium that was sufficient to kill ~50% of algae within 24 h (Supporting Fig. 1). We started the algal cultures with a seeding inoculate to reach chlorophyll *a* 0.1 RFU × 10^4^ and cultivated them for ~5 days until mid-exponential phase (Chl *a* ca. 1.0 RFU × 10^4^). Then, the cultures were treated with the bacterial CFSM and incubated for 24 h, which resulted in the lysis of approximately half of the cells. The cultures containing the surviving cells were used to inoculate fresh medium to reach again a chlorophyll *a* fluorescence of 0.1 RFU × 10^4^, thereby terminating a cycle (Fig. 1). Culturing/lysis cycles were repeated and the evolved cultures were tested for increased resistance compared to the seeding stock cultures.

**Figure 2:**
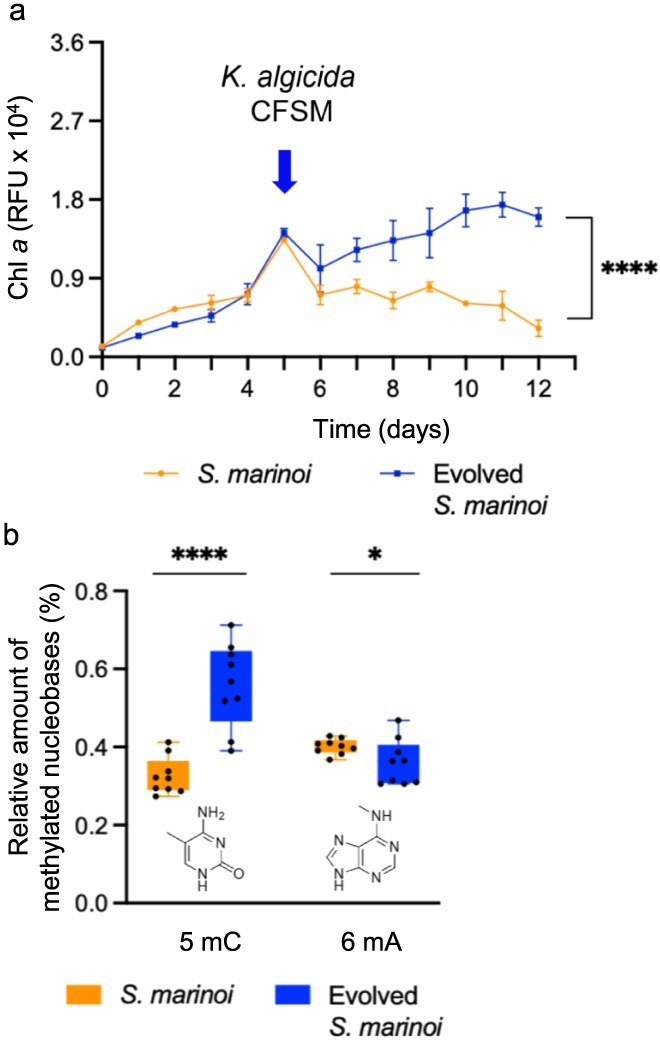
Growth curve alteration after exposure to algicidal spent medium and DNA methylation of control and evolved *S. marinoi*. (a) *Skeletonema marinoi* evolved for 11 cycles grows significantly better after addition of CFSM from *K. algicida* compared to the control. The untreated cultures reached Chl *a* of ca. 2.7 RFU × 10^4^ after 12 days (see Supporting Fig. 1). Error bars represent standard deviation (biological replicates, *N* = 3), the unpaired *t-*test confirms statistical significance (*P*-value: ****P < .0001, day 13). (b) DNA methylation, here depicted as 5-methylcytosine (5mC) and 6-methyladenine (6mA) amounts relative to the total unmethylated bases, significantly differs in control and evolved *S. marinoi*. Error bars represent standard deviation (three biological replicates, with each three technical replicates). Statistical analysis is based on Kruskal–Wallis ANOVA (*P*-value: *P < .05 and ****P < .0001). Box plots were created using GraphPad prism v.10.

Already after 11 cycles, we observed a significantly increased resistance of evolved *S. marinoi* toward CFSM compared to the original strain (Fig. 2a). The growth curve of *S. marinoi* and evolved *S. marinoi* (cycle-11) without the addition of CFSM is not significantly different, showing that the evolved response is not affecting other growth parameters (Supporting Fig. 2). Comparable rapid evolutionary responses were reported for other phytoplankton cells in previous studies. The diatom *Phaeodactylum tricornutum* showed enhanced biomass production, fucoxanthin accumulation, and increased all-*trans* β-carotene and lutein content compared to the initial strain if selected under photo-oxidative stress for 11 cycles [30]. The evolved green phytoplankton *Dunaliella salina* showed enhanced light tolerance under red LED illumination after ALE for 16 cycles [31]. Thus, our results confirm that phenotype changes in evolved phytoplankton can manifest rapidly.

Continuous long-term exposure to environmental changes can lead to modifications in DNA methylation in diatoms, which can be transferred over generations, resulting in inheritable changes in the physiological functions of algae [32]. The methylation of cytosine to 5-methyl cytosine (5mC) and of adenine to 6-methly adenine (6mA) are well-known epigenetic markers associated with transcriptional regulation in diatoms [33]. Here, we observed a pronounced increase in 5mC but a reduction of 6mA levels in the evolved strain compared to the control (Fig. 2b). The observed methylation levels in evolved and control cultures are well in accordance with those reported from diatoms in previous studies (0.1% to 2.2% 5mC) [34]. The evolved diatoms in our study thus might maintain heritable plasticity that does not rely on changes in DNA sequence which is in accordance with an epigenetic regulation of resistance in *S. marinoi.* Such epigenetic processes may play a significant role in mediating responses to the highly dynamic and unpredictable environmental conditions these organisms live in.

### Untargeted metabolomics reveals candidate chemical mediators of resistance

**Figure 3:**
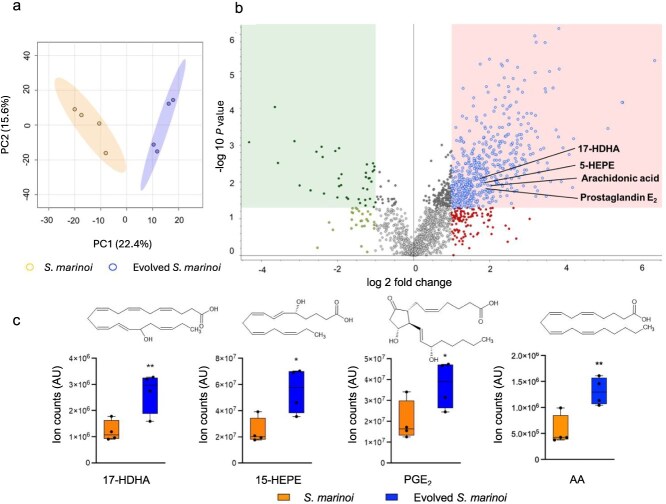
Untargeted comparative metabolomics-based identification of candidate metabolites responsible for the evolved *S. marinoi* resistance against *K. algicida*. (a) Susceptible *S. marinoi* and evolved *S. marinoi* cycle-11 have a distinct cellular metabolome as evidenced in the PCA score plot. The ellipses represent the 95% confidence region. (b) The volcano plot comparing evolved *S. marinoi* and susceptible *S. marinoi* allows to identify the features (i.e. mass/retention time pairs) that were significantly altered between evolved and control *S. marinoi*. The dots in the right field represent features with statistical significance *P* <.05 and a greater than 2-fold up-regulation in evolved algae; the dots in the left field show significantly down-regulated features. (c) Bar graphs for oxylipins (5-HEPE, 17-HDHA, PGE_2_) and AA. These compounds are significantly up-regulated in evolved *S. marinoi* compared to controls. Box plots illustrate the intensity changes of the four selected chemical mediators in evolved and control *S. marinoi*. The integrated ion counts are given in arbitrary units (AU). Two-tailed Student’s *t-*test was used to determine the significance (ns *P* > .05, *P ≤ .05, **P ≤ .01, biological replicates *N* = 4). Box plots were created using GraphPad prism v.10.

We studied the evolved intracellular metabolic response of *S. marinoi* using an untargeted comparative metabolomics comparison of evolved *S. marinoi* (cycle-11) and susceptible *S. marinoi*. The survey was conducted after extraction with a solvent mixture optimized to obtain broad metabolic coverage. Analysis was carried out on an ultra-high-performance liquid chromatography–high-resolution mass spectrometer (Orbitrap UHPLC–HRMS) using a C18 column for separation. We analyzed the metabolomics data using the software Compound Discoverer*.* Peak intensities were normalized to the cell densities (Supporting Table 1), yielding a matrix with 1651 signals (molecular formula, *m/z*, retention time). Endometabolome profiles of extracts from control *S. marinoi* and evolved *S. marinoi* cells were clearly discriminated by PCA with a total principal component variance of 40.3%; the 95% confidence ellipses do not overlap, suggesting strong group separation (Fig. 3a). The volcano plot allowed us to identify significantly regulated features (Fig. 3b).

Based on the mass spectra of elevated features in evolved strain extracts, 29 metabolites were tentatively elucidated with high-resolution MS/MS, of which 4 were further confirmed with authentic standards. Among the most significantly up-regulated metabolites in evolved *S. marinoi* on cyle-11 were the oxylipins 5-hydroxyeicosapentaenoic acid (5-HEPE) at *m/z* 317.2131 [M-H]^−^, 17-hydroxydocosahexaenoic acid (17-HDHA) at *m/z* 343.2279 [M-H]^−^, prostaglandin E_2_ (PGE_2_), *m/z* 351.2182 [M-H]^−^, and the fatty acid arachidonic acid (AA) *m/z* 303.2351 [M-H]^−^ (structures were confirmed by comparison with analytical standards, Supporting Figs 3–8). The (*R*) stereochemistry of 5-HEPE was determined on a modified cellulose column with an acetonitrile/water gradient in accordance with the reported elution order (Supporting Fig. 9) [35].

Hydroxylated polyunsaturated fatty acids are widespread in diatoms and can be linked to their chemical defense [10, 36]. The AA-derived prostaglandin E_2_ is a well-known mammalian lipid mediator involved in ovulation, bone metabolism, blood vessel tone, and pain and has also been detected in red algae where it acts as a defensive metabolite [37]. In *S. marinoi,* up-regulated prostaglandin levels were detected under herbivore pressure [38].

**Figure 4:**
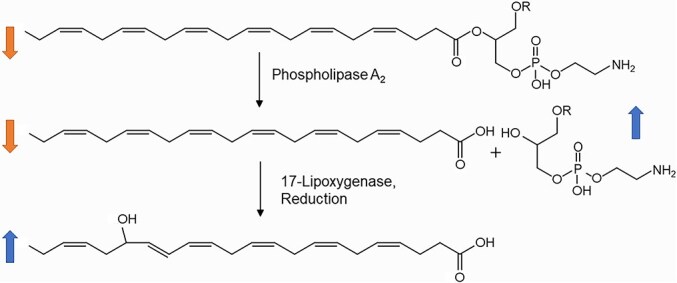
The putative pathways of oxylipin formation based on regulated features in the evolved *S. marinoi* metabolome. Phospholipids (R = fatty acid) are cleaved by phospholipases resulting in the release of free precursor fatty acids (depicted here is DHA, released fatty acids also include EPA and ARA). These fatty acids are transformed by lipoxygenases and further downstream enzymes to the respective oxylipins. DHA is the precursor of 17-HDHA as depicted here, and EPA can be transformed to 5-HETE and ARA to prostaglandin E_2_. Although oxylipins and lysolipids are up-regulated reaction products, the precursor lipids and fatty acids are down-regulated.

The precursors of 5-HEPE and 17-HDHA, namely, eicosapentaenoic acid (EPA) at *m/z* 301.2144 [M-H]^−^ and docosahexaenoic acid (DHA) at *m/z* 327.2298 [M-H]^−^, were down-regulated in evolved cultures, consistent with their further transformation to the oxylipins. Also, the putative phospholipids, phosphatidylethanolamine (PE (22:6/20:5)) and PE (20:5/18:1) at *m/z* 808.4911 [M-H]^−^ and *m/z* 762.5075 [M-H]^−^, respectively, were down-regulated in evolved *S. marinoi*, indicating that these might act as precursors from which phospholipases can liberate the fatty acid substrates (Fig. 4) [39, 40]. In agreement with this pathway, the putative lysophospholipids were up-regulated in resistant strains. These include PEs, namely, lyso PE (20:5), lyso PE (22:6), and lyso PE (20:4) at *m/z* 498.2620 [M-H]^−^, *m/z* 524.2778 [M-H]^−^, and *m/z* 560.2990 [M-H]^−^, respectively (Supporting Table 2). The regulation patterns of these lysolipids are consistent with an enzymatic release of free fatty acids from phospholipids, followed by their oxidative lipoxygenase-mediated transformation (Fig. 4). Such a reaction sequence is also known from the oxylipin-based wound-activated chemical defense of diatoms against herbivores [39, 40]. Unexpectedly, arachidonic acid, the precursor of PGE_2_, is up-regulated in evolved cultures. This might be explained with the fact that the multi-step PGE_2_ biosynthesis is not substrate limited as it is the case with the rapid lipoxygenase-mediated transformations to 5-HEPE and 17-HDHA [28]. If all three PUFAs (EPA, DHA, and AA) are released from storage lipids and further transformed, the different kinetics of these transformations might result in the observed pattern.

### Oxylipins mediate resistance against algicidal bacteria

**Figure 5:**
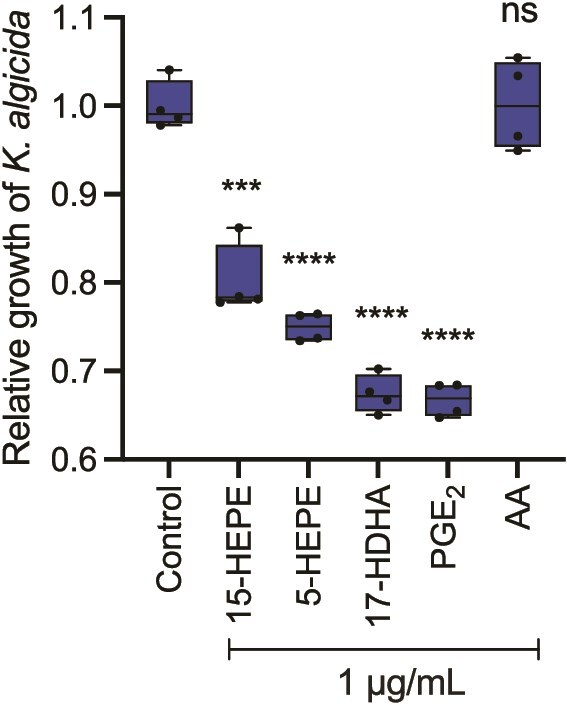
Effect of oxylipins (5-HEPE, 17-HDHA, PGE_2_) and the fatty acid AA on the growth of *K. algicida* (1 μg ml^−1^, mean ± SD, biological replicates, *N* = 4). Growth was monitored by following the OD 550 after 24 h and normalized to the solvent control (no added compounds). 15-HEPE was used as a positive control [10]. Statistically significant differences (unpaired *t-*test) between control cultures and cultures treated with *K. algicida* are shown as *P*-value: ***P < .001, ****P < .0001, ns: not significant. Box plots were created by GraphPad prism v.10.

To verify if the wound-activated formation of oxylipins constitutes a defense mechanism of the alga against the bacterial pathogen, we evaluated the effect of the identified C20 oxylipins 5-HEPE and PGE_2_, the C22 oxylipin 17-HDHA, 15-HEPE, and AA on the bacterium *K. algicida*. All four oxylipins inhibit the growth of the bacteria with an effective concentration of 1 μg ml^−1^, with PGE_2_ being most effective; AA was inactive when exposed to algicidal bacteria (Fig. 5). This concentration is still below the estimated production of oxylipins by diatoms [10] even if concentrations of oxylipins from diatoms can vary substantially. The oxylipins thus may contribute to the evolved chemical defense of the alga against *K. algicida*. This is supported by the observation that the structurally related 15-HEPE is formed constitutively in the diatom *C. didymus* and acts as inhibitor of *K. algicida* growth [10, 14]. Some diatoms, which are resistant against *K. algicida*, thus rely on similar defenses compared to those evolved by the initially susceptible *S. marinoi*. Whether *S. marinoi* might only evolve these defenses in case of re-occurring pathogen pressure has to be established in future field experiments.

In contrast to the oxylipins that all exhibited activity, the free fatty acid is not active. The targets of oxylipins in bacteria are not yet identified, and our study does not reveal any specific structural element because PGE_2_ as well as simple hydroxylated fatty acids are active. In many organisms, including marine microalgae, oxylipins are known to function as infochemicals or damage-associated molecular patterns, capable of triggering intracellular signaling cascades related to stress response, programmed cell death, or defense activation [41]. Our study adds now the aspect that not only damage but also sublethal exposure to harmful factors can result in the evolution of an oxylipin-based defense.

Integrating both the ALE approach and metabolomics has successfully revealed chemical defense and evolutionary trajectories in phytoplankton–bacteria interactions. The results presented here are derived from laboratory experiments, and implications for plankton ecology can only be speculative. They, however, provide a general understanding how *S. marinoi* adapts to and survives to threats it encounters in its environment. It will be interesting to evaluate how the multiple simultaneous threats the alga encounters in complex plankton communities will allow an adaptive evolutionary response. Given the vast abundance and central role of diatoms in marine ecosystems, the results presented here have implications for different disciplines including ecological forecasting, fishery yield prediction, and climate modeling.

## Supplementary Material

Azizah_et_al__Supplementary_information_for_production_wrag111

## Data Availability

All raw files and metabolomic datasets from algal extracts (*S. marinoi* RCC75 and evolved *S. marinoi*) are publicly available in the MassIVE, GNPS [42] public repository, under accession number MSV000099304. Reference MS/MS spectra of 5-HEPE, 17-HDHA, PGE_2_, arachidonic acid, eicosapentaenoic acid (EPA), and docosahexaenoic acid (DHA) were deposited in the GNPS public repository under accession numbers CCMSLIB00013560084, CCMSLIB00015179645, CCMSLIB00015598037, CCMSLIB00014901994, CCMSLIB00015078977, and CCMSLIB00015088248, respectively.

## References

[1] Deng Y, Vallet M, Pohnert G. Temporal and spatial signaling mediating the balance of the plankton microbiome. *Annu Rev Mar Sci* 2022;14:239–60. 10.1146/annurev-marine-042021-012353 34437810

[2] Field CB, Behrenfeld MJ, Randerson JT et al. Primary production of the biosphere: integrating terrestrial and oceanic components. *Science* 1998;281:237–40. 10.1126/science.281.5374.237 9657713

[3] Falkowski PG, Barber RT, Smetacek V. Biogeochemical controls and feedbacks on ocean primary production. *Science* 1998;281:200–6. 10.1126/science.281.5374.200 9660741

[4] Wang H, Chen F, Mi T et al. Responses of marine diatom *Skeletonema marinoi* to nutrient deficiency: programmed cell death. *Appl Environ Microbiol* 2020;86:02460. 10.1128/AEM.02460-19 PMC6974647 31757826

[5] Johansson ON, Pinder MIM, Ohlsson F et al. Friends with benefits: exploring the phycosphere of the marine diatom *Skeletonema marinoi*. *Front Microbiol* 2019;10:01828. 10.3389/fmicb.2019.01828 PMC6691348 31447821

[6] Sohn JH, Lee JH, Yi H et al. *Kordia algicida* gen. nov., sp nov., an algicidal bacterium isolated from red tide. *Int J System Evol Microbiol* 2004;54:675–80. 10.1099/ijs.0.02689-0 15143006

[7] Paul C, Pohnert G. Interactions of the algicidal bacterium *Kordia algicida* with diatoms: regulated protease excretion for specific algal lysis. *PLoS One* 2011;6:e21032. 10.1371/journal.pone.0021032 21695044 PMC3117869

[8] Bigalke A, Meyer N, Papanikolopoulou LA et al. The algicidal bacterium *Kordia algicida* shapes a natural plankton community. *Appl Environ Microbiol* 2019;85:e02779–18. 10.1128/AEM.02779-18 PMC6585488 30737345

[9] Bigalke A, Pohnert G. Algicidal bacteria trigger contrasting responses in model diatom communities of different composition. *MicrobiologyOpen* 2019;8:e00818. 10.1002/mbo3.818 30809963 PMC6692526

[10] Meyer N, Rettner J, Werner M et al. Algal oxylipins mediate the resistance of diatoms against algicidal bacteria. *Mar Drugs* 2018;16:486. 10.3390/md16120486 30518148 PMC6315584

[11] Meyer N, Pohnert G. Isolate-specific resistance to the algicidal bacterium *Kordia algicida* in the diatom *Chaetoceros genus*. *Bot Marina* 2019;62:527–35. 10.1515/bot-2019-0007

[12] Wichard T, Beemelmanns C. Role of chemical mediators in aquatic interactions across the prokaryote-eukaryote boundary. *J Chem Ecol* 2018;44:1008–21. 10.1007/s10886-018-1004-7 30105643

[13] Pancic M, Kiorboe T. Phytoplankton defence mechanisms: traits and trade-offs. *Biol Rev* 2018;93:1269–303. 10.1111/brv.12395 29356270

[14] Paul C, Mausz MA, Pohnert G. A co-culturing/metabolomics approach to investigate chemically mediated interactions of planktonic organisms reveals influence of bacteria on diatom metabolism. *Metabolomics* 2013;9:349–59. 10.1007/s11306-012-0453-1

[15] Syhapanha KS, Russo DA, Deng Y et al. Transcriptomics-guided identification of an algicidal protease of the marine bacterium *Kordia algicida* OT-1. *MicrobiologyOpen* 2023;12:e1387. 10.1002/mbo3.1387 37877654 PMC10565126

[16] Jebali A, Sanchez MR, Hanschen ER et al. Trait drift in microalgae and applications for strain improvement. *Biotechnol Adv* 2022;60:108034. 10.1016/j.biotechadv.2022.108034 36089253

[17] Schlüter L, Lohbeck KT, Gröger JP et al. Long-term dynamics of adaptive evolution in a globally important phytoplankton species to ocean acidification. *Sci Adv* 2016;2:e1501660. 10.1126/sciadv.1501660 27419227 PMC4942326

[18] Lohbeck KT, Riebesell U, Reusch TBH. Adaptive evolution of a key phytoplankton species to ocean acidification. *Nat Geosci* 2012;5:346–51. 10.1038/ngeo1441

[19] Perrineau MM, Zelzion E, Gross J et al. Evolution of salt tolerance in a laboratory reared population of *Chlamydomonas reinhardtii*. *Environ Microbiol* 2014;16:1755–66. 10.1111/1462-2920.12372 24373049

[20] Huang RP, Ding JC, Gao KS et al. A potential role for epigenetic processes in the acclimation response to elevated pCO_2_ in the model diatom *Phaeodactylum tricornutum*. *Front Microbiol* 2019;9:3342. 10.3389/fmicb.2018.03342 30692981 PMC6340190

[21] Maier I, Calenberg M. Effect of extracellular Ca^2+^ and Ca^2+^-antagonists on the movement and chemoorientation of male gametes of *Ectocarpus siliculosus* (Phaeophyceae). *Bot Acta* 1994;107:451–60. 10.1111/j.1438-8677.1994.tb00820.x

[22] Azizah M, Pohnert G. 2-Homoectoine: an additional member of the ectoine family from phyto- and bacterioplankton involved in osmoadaptation. *J Nat Prod* 2023;87:50–7. 10.1021/acs.jnatprod.3c00766 38150306

[23] Vallet M, Baumeister TUH, Kaftan F et al. The oomycete *Lagenisma coscinodisci* hijacks host alkaloid synthesis during infection of a marine diatom. *Nat Commun* 2019;10:4938. 10.1038/s41467-019-12908-w 31666506 PMC6821873

[24] Cooper B, Yang R. An assessment of acquireX and compound discoverer software 3.3 for non-targeted metabolomics. *Sci Rep* 2024;14:4841. 10.1038/s41598-024-55356-3 38418855 PMC10902394

[25] Ghaderiardakani F, Langhans L, Kurbel VB et al. Metabolite profiling reveals insights into the species-dependent cold stress response of the green seaweed holobiont Ulva (Chlorophyta). *Env Exp Botany* 2022;200:104913. 10.1016/j.envexpbot.2022.104913

[26] Dunn WB, Broadhurst D, Begley P et al. Procedures for large-scale metabolic profiling of serum and plasma using gas chromatography and liquid chromatography coupled to mass spectrometry. *Nature Prot* 2011;6:1060–83. 10.1038/nprot.2011.335 21720319

[27] Pang ZQ, Lu Y, Zhou GY et al. MetaboAnalyst 6.0: towards a unified platform for metabolomics data processing, analysis and interpretation. *Nucleic Acids Res* 2024;52:W398–406. 10.1093/nar/gkae253 38587201 PMC11223798

[28] Werner M, Jordan PM, Romp E et al. Targeting biosynthetic networks of the proinflammatory and proresolving lipid metabolome. *FASEB J* 2019;33:6140–53. 10.1096/fj.201802509R 30735438 PMC6988863

[29] Otto JFM, Pohnert G, Wichard T et al. Global DNA-methylation in quantitative epigenetics: orbitrap mass spectrometry. *Fornt Mol Biosci* 2025;12:1681568. 10.3389/fmolb.2025.1681568 PMC12511803 41078603

[30] Yi ZQ, Xu MN, Magnusdottir M et al. Photo-oxidative stress-driven mutagenesis and adaptive evolution on the marine diatom *Phaeodactylum tricornutum* for enhanced carotenoid accumulation. *Mar Drugs* 2015;13:6138–51. 10.3390/md13106138 26426027 PMC4626683

[31] Fu WQ, Guomundsson O, Paglia G et al. Enhancement of carotenoid biosynthesis in the green microalga *Dunaliella salina* with light-emitting diodes and adaptive laboratory evolution. *Appl Microbiol Biotechnol* 2013;97:2395–403. 10.1007/s00253-012-4502-5 23095941 PMC3586100

[32] Maumus F, Rabinowicz P, Bowler C et al. Stemming epigenetics in marine stramenopiles. *Cur Genomics* 2011;12:357–70. 10.2174/138920211796429727 PMC3145265 22294878

[33] Hoguin A, Yang F, Groisillier A et al. The model diatom *Phaeodactylum tricornutum* provides insights into the diversity and function of microeukaryotic DNA methyltransferases. *Commun Biol* 2023;6:253. 10.1038/s42003-023-04629-0 36894681 PMC9998398

[34] Jarvis EE, Dunahay TG, Brown LM. DNA nucleoside composition and methylation in several species of microalgae. *J Phycol* 1992;28:356–62. 10.1111/j.0022-3646.1992.00356.x

[35] Cebo M, Fu XQ, Gawaz M et al. Enantioselective ultra-high performance liquid chromatography-tandem mass spectrometry method based on sub-2μm particle polysaccharide column for chiral separation of oxylipins and its application for the analysis of autoxidized fatty acids and platelet releasates. *J Chromatogr A* 2020;1624:461206. 10.1016/j.chroma.2020.461206 32540064

[36] Miralto A, Barone G, Romano G et al. The insidious effect of diatoms on copepod reproduction. *Nature* 1999;402:173–6. 10.1038/46023

[37] Jagusch H, Werner M, Werz O et al. 15-Hydroperoxy-PGE(2): intermediate in mammalian and algal prostaglandin biosynthesis. *Angew Chem Int Edit* 2019;58:17641–5. 10.1002/anie.201910461 PMC6899959 31529599

[38] Barbarinaldi R, Di Costanzo F, Orefice I et al. Prostaglandin pathway activation in the diatom *Skeletonema marinoi* under grazer pressure. *Mar Environ Res* 2024;196:106395. 10.1016/j.marenvres.2024.106395 38382127

[39] Pohnert G . Wound-activated chemical defense in unicellular planktonic algae. *Angew Chem Int Edit* 2000;39:4352–4. 10.1002/1521-3773(20001201)39:23<4352::AID-ANIE4352>3.0.CO;2-U 29711885

[40] Pohnert G . Phospholipase a(2) activity triggers the wound-activated chemical defense in the diatom *Thalassiosira rotula*. *Plant Physiol* 2002;129:103–11. 10.1104/pp.010974 12011342 PMC155875

[41] Jagusch H, Baumeister TUH, Pohnert G. Mammalian-like inflammatory and pro-resolving oxylipins in marine algae. *ChemBioChem* 2020;21:2419–24. 10.1002/cbic.202000178 32239741 PMC7496315

[42] Wang MX, Carver JJ, Phelan VV et al. Sharing and community curation of mass spectrometry data with global natural products social molecular networking. *Nature Biotechnol* 2016;34:828–37. 10.1038/nbt.3597 27504778 PMC5321674

