# Supplementary material for: Rapid adaptive evolution of oxylipin-based chemical defense against algicidal bacteria in a bloom-forming diatom
Source: ISME J. 2026 May 8;20(1):wrag111. doi: 10.1093/ismejo/wrag111 (PMC13240593; doi:10.1093/ismejo/wrag111)
Supplement: Azizah_et_al__Supplementary_information_for_production_wrag111 [file azizah_et_al__supplementary_information_for_production_wrag111.docx]

**Supplemental Material**

**Rapid adaptive evolution of oxylipin-based chemical defence against algicidal bacteria in a bloom-forming diatom**

Muhaiminatul Azizah^1^, Janine F. M. Otto^[1]^, Nico Ueberschaar^[2]^, Markus Werner^[3]^, Oliver Werz^[3]^, Georg Pohnert*^[1]^

^[1]^ Dr. Muhaiminatul Azizah, M.Sc. Janine F. M. Otto, Prof. Dr. Georg Pohnert Bioorganic Analytics, Institute for Inorganic and Analytical Chemistry, Friedrich Schiller University, Lessingstrasse 8, D-07743 Jena, Germany

^[2]^ Dr. Nico Ueberschaar Mass Spectrometry Platform, Friedrich Schiller University Jena, Humboldtstr. 8, 07743 Jena, Germany

^[3]^ Dr. Markus Werner, Prof. Dr. Oliver Werz, Department of Pharmaceutical/Medicinal Chemistry, Institute of Pharmacy, Friedrich Schiller University Jena, 07743 Jena, Germany

*Corresponding author email [Georg.Pohnert@uni-jena.de](mailto:Georg.Pohnert@uni-jena.de)


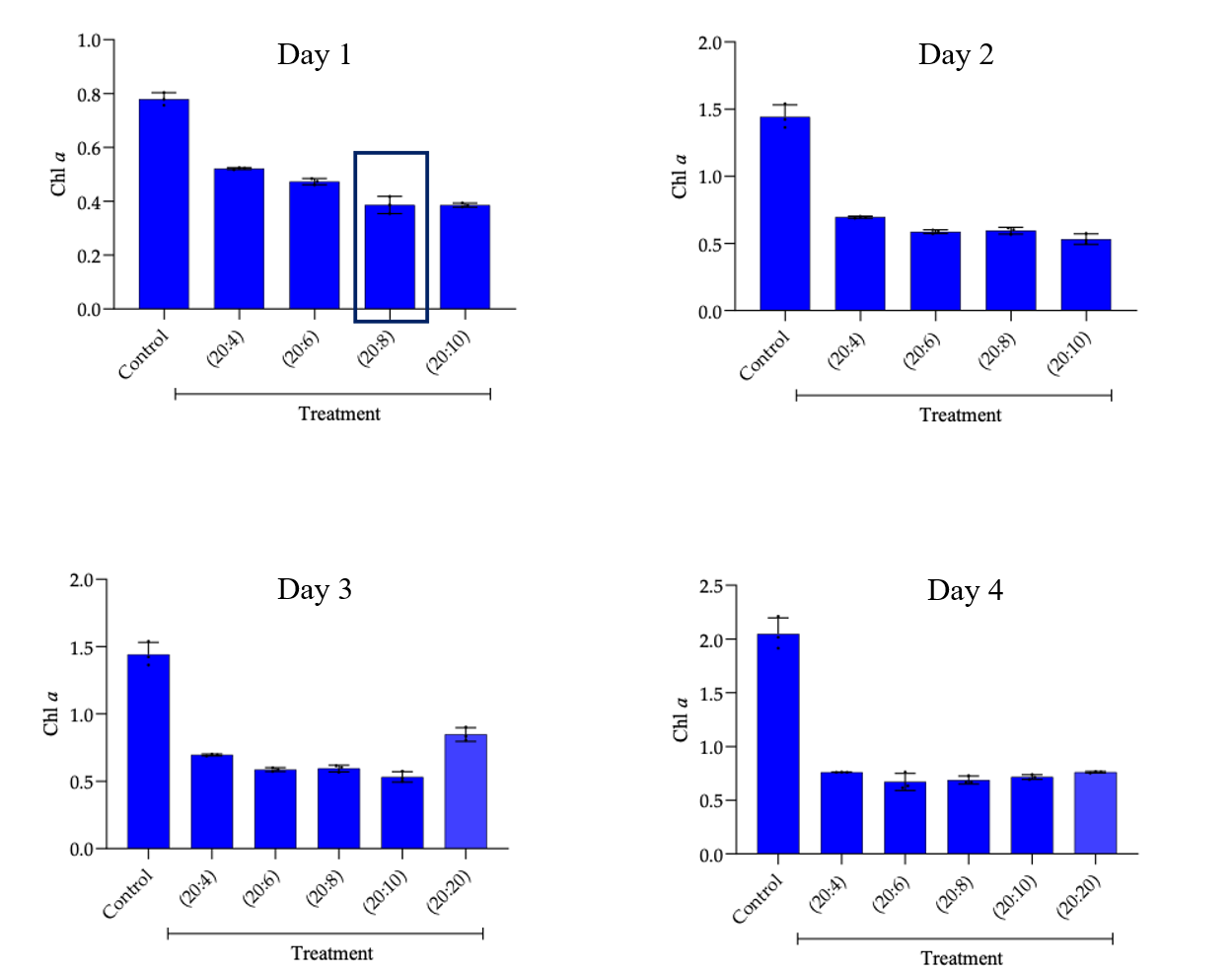


Supporting Figure 1. Preliminary lysis study by cell-free spent medium (CFSM) from the bacterial culture compared to a medium control. The medium control consisted of marine broth:ASW (1:20 *v*/*v*). The ratio of *S. marinoi* cultures and CFSM are given for the treatments. Note: Values for the 20:20 dilution were only taken at days 3 and 4.


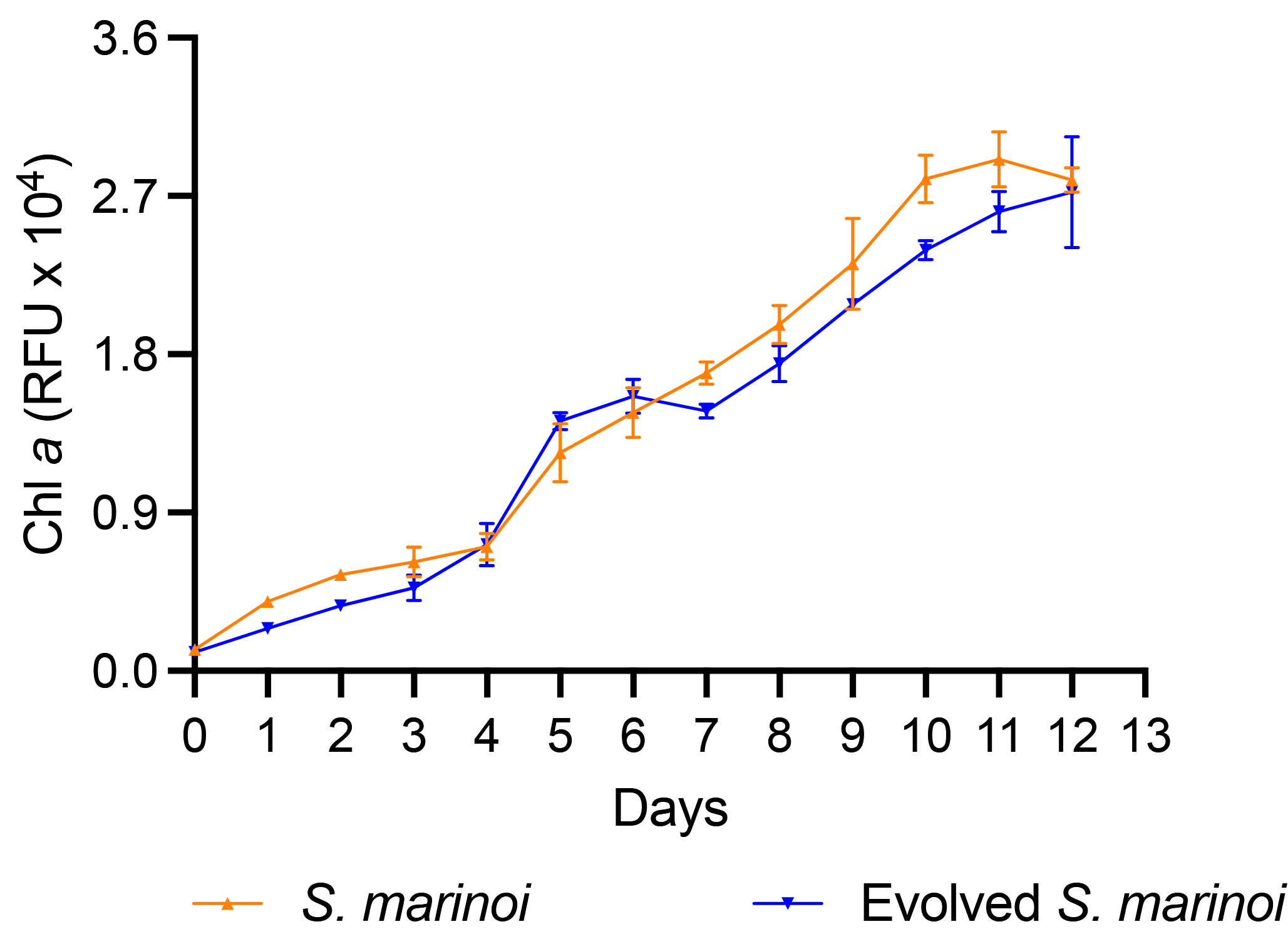


**Supporting Figure 2.** Growth curve of phytoplankton *S. marinoi* and evolved *S. marinoi* obtained after cycle-11 without adding cell-free spent medium.

**Supporting Figure 3.** MS/MS of identified 5-hydroxyeicosapentaenoic acid (5-HEPE) from the evolved *S. marinoi* extract compared to the analytical standard. Retention times of the analyte and the standard matched.


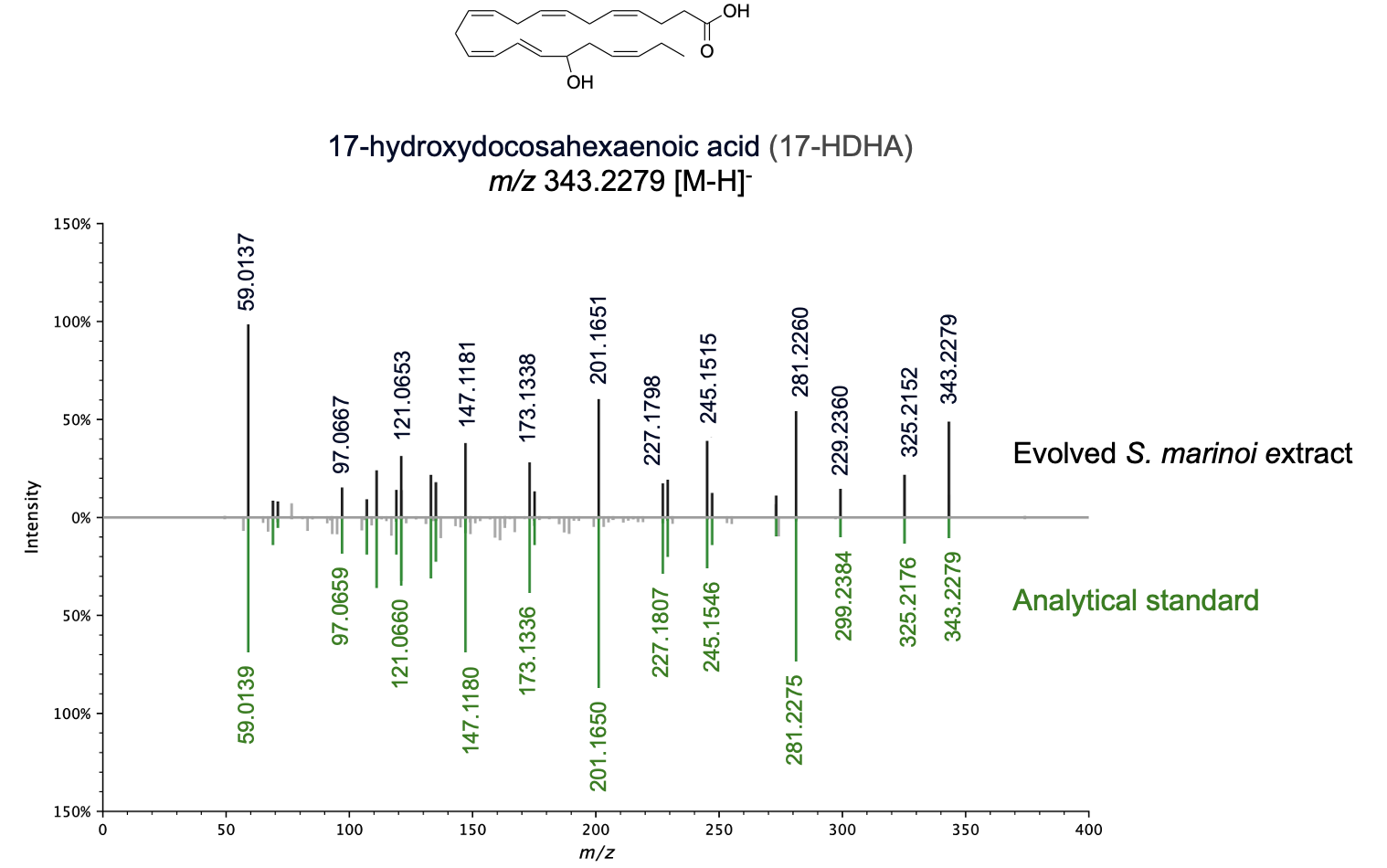


**Supporting Figure 4** MS/MS of identified 17-hydroxydocosahexaenoic acid (17-HDHA) from the evolved *S. marinoi* extract compared to the analytical standard. Retention times of the analyte and the standard matched.


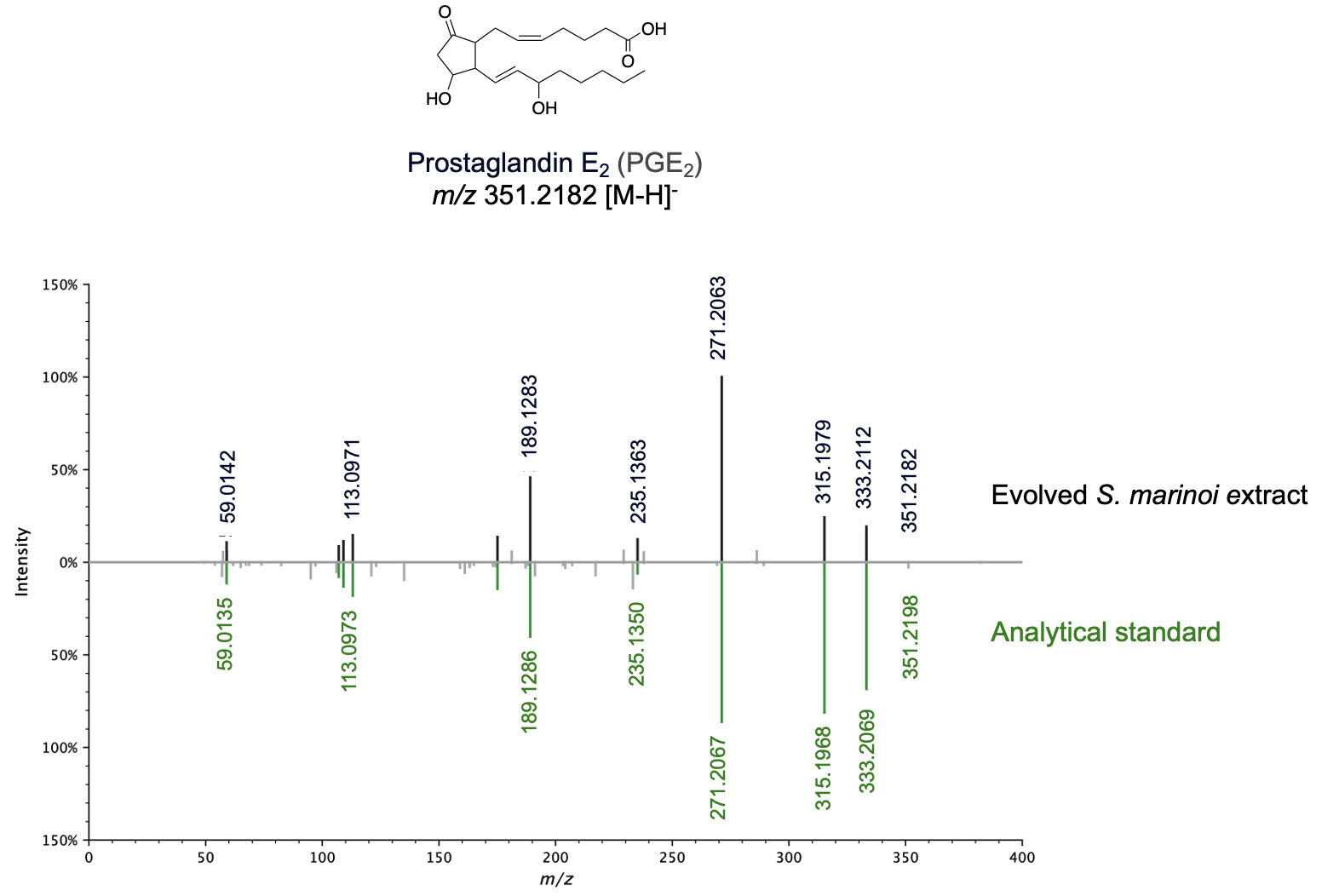


**Supporting Figure 5.** MS/MS of identified prostaglandin E_2_ (PGE_2_) from the evolved

*S. marinoi* extract compared to the analytical standard. Retention times of the analyte and the standard matched.


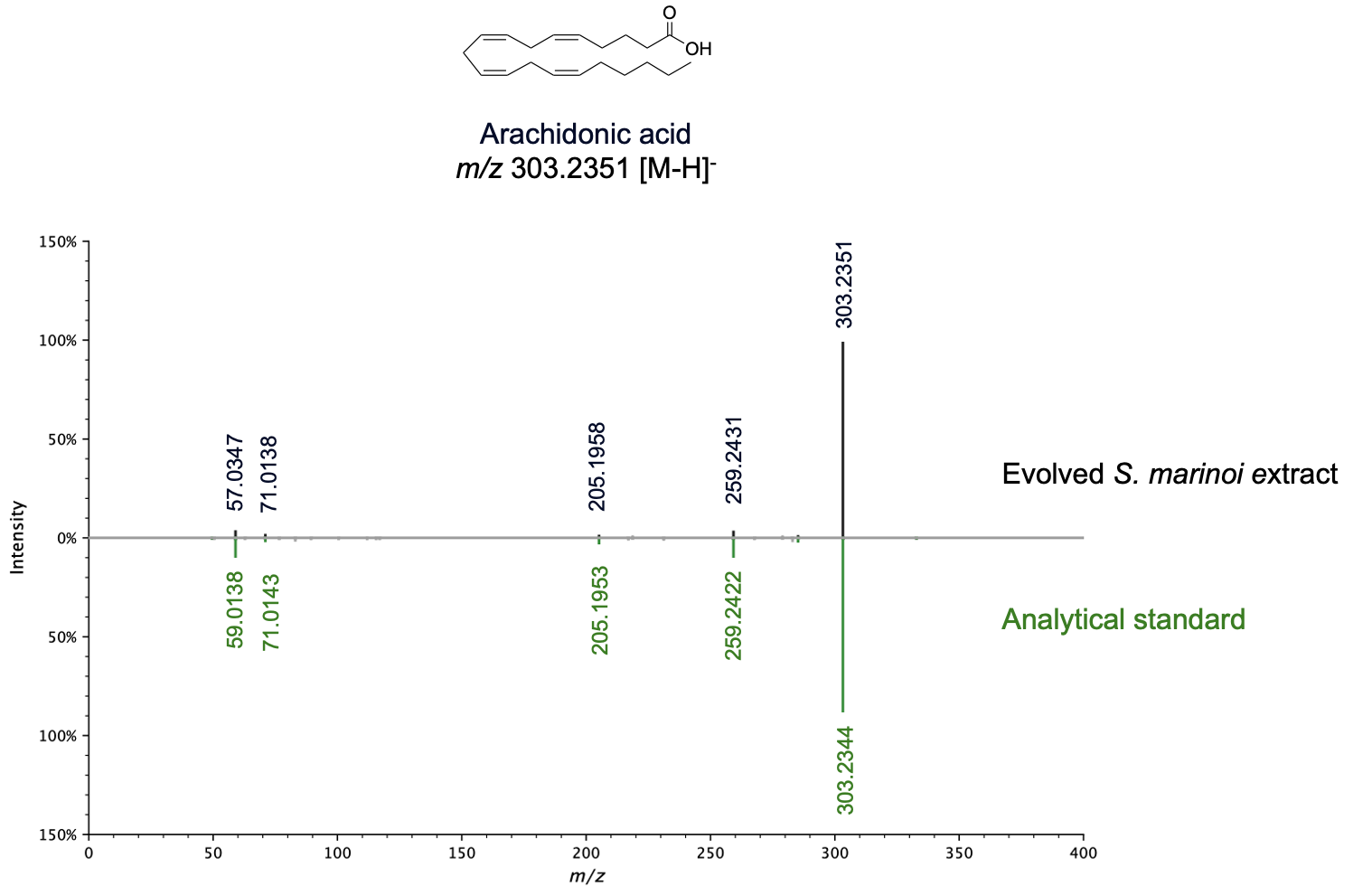


**Supporting Figure 6.** MS/MS of identified arachidonic acid (AA) from the evolved

*S. marinoi* extract compared to the analytical standard. Retention times of the analyte and the standard matched.


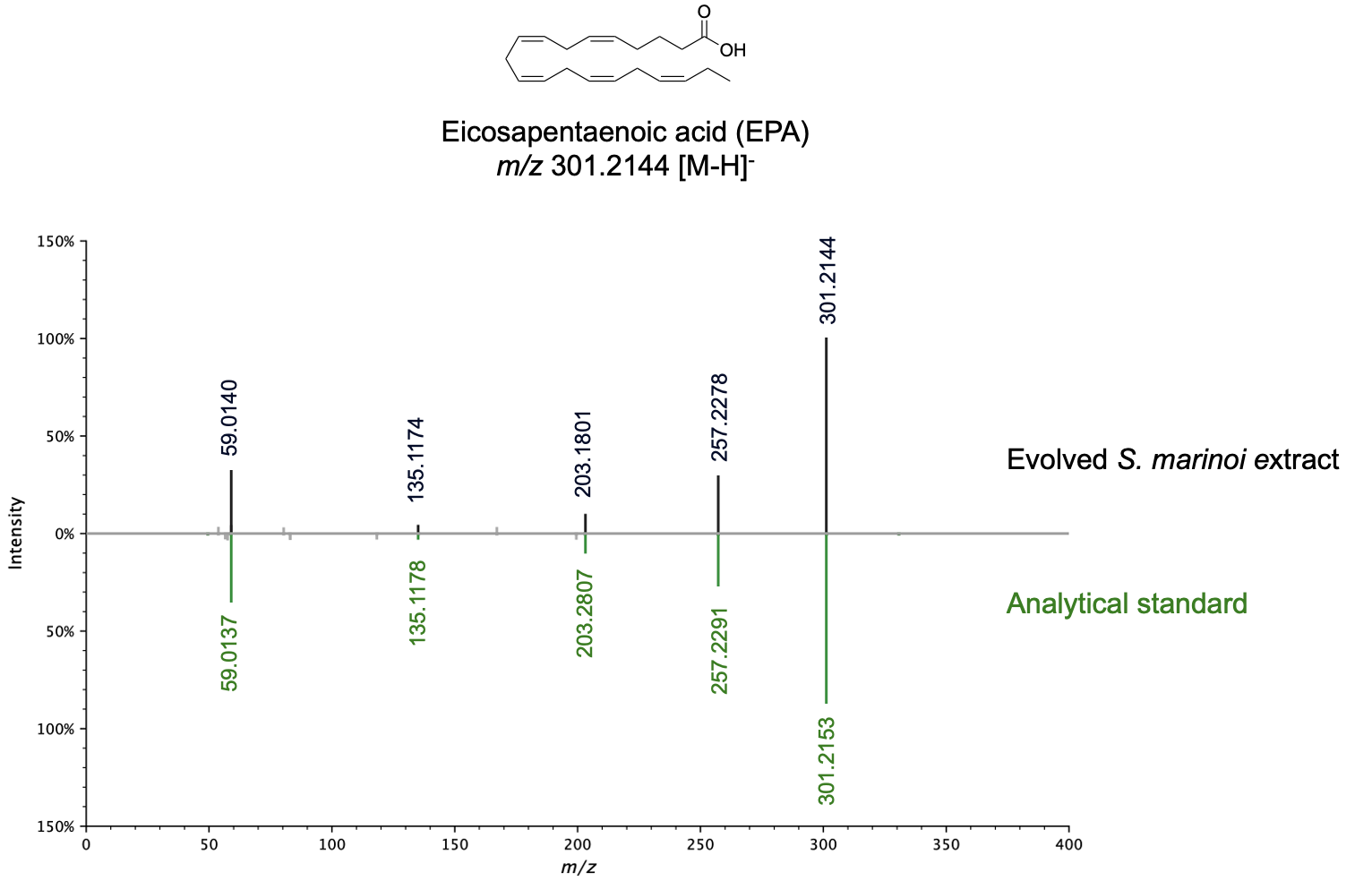


**Supporting Figure 7.** MS/MS of identified eicosapentaenoic acid (EPA) from the evolved *S. marinoi* extract compared to the analytical standard. Retention times of the analyte and the standard matched.


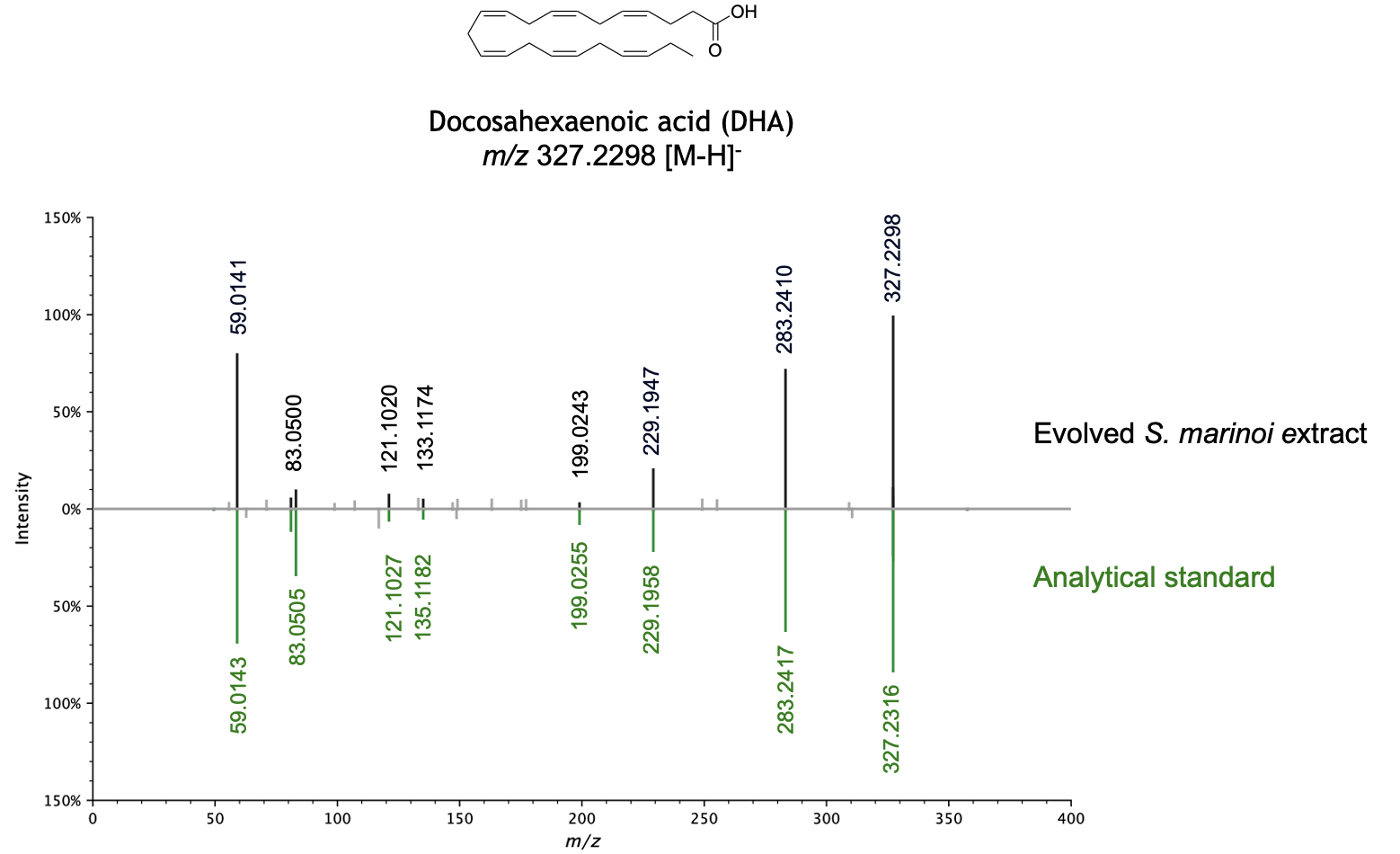


**Supporting Figure 8.** MS/MS of identified docosahexaenoic acid (DHA) from the evolved *S. marinoi* extract compared to the analytical standard. Retention times of the analyte and the standard matched.


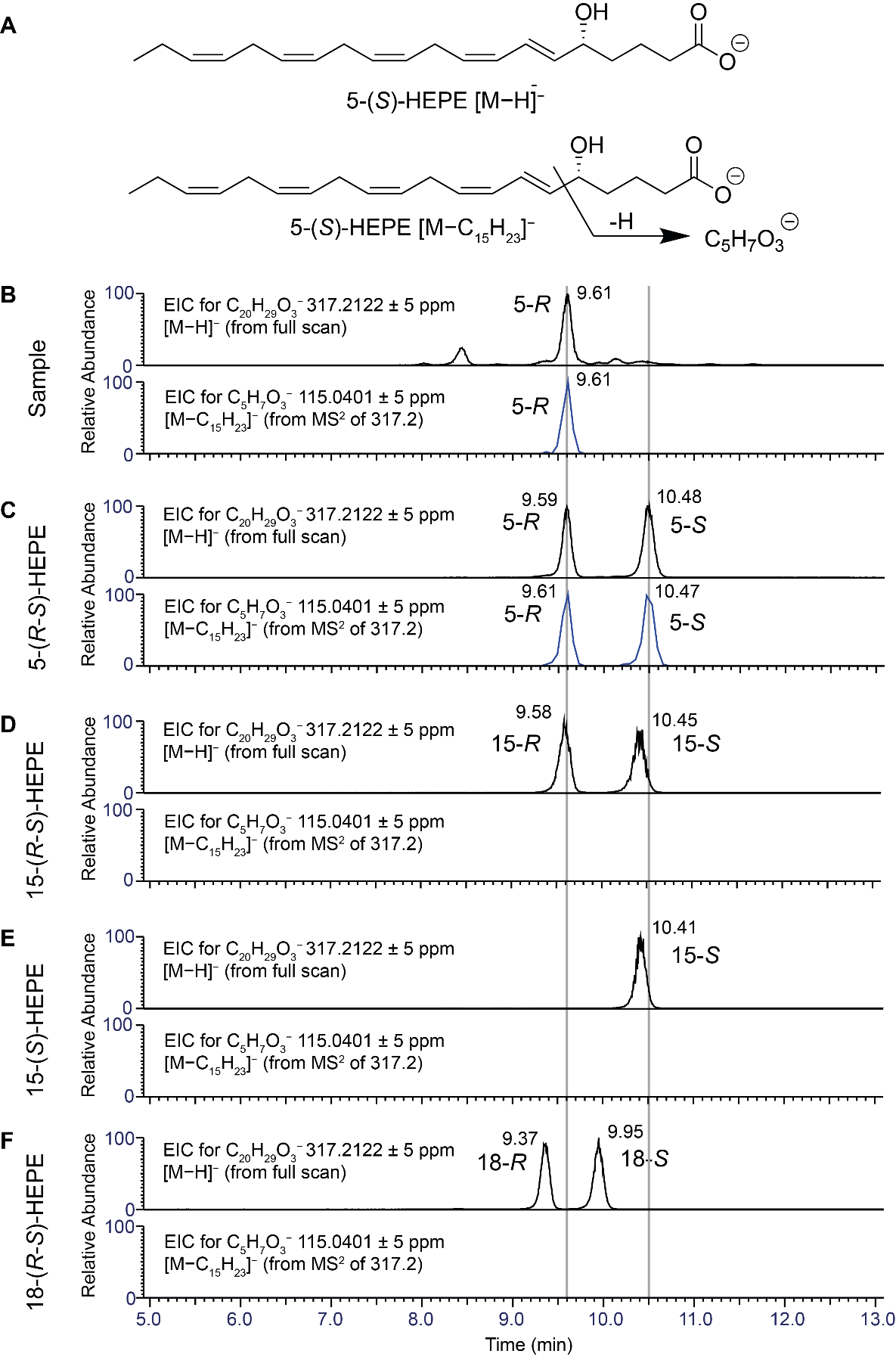


**Supporting Figure 9.** Chiral separation of 5-HEPE. **A**: Fragmentation of 5-HEPE and a key fragment (15 NCE) which is crucial for regio chemistry of the hydroxy group. **B**-**F**: plot of the chromatograms. On top of each figure part the EIC of the deprotonated molecule and on the bottom the EIC of the fragment named in figure part **A**. **B**: sample, **C**: 5-(***R***-***S***)-HEPE, **D**: 15-(*R*-*S*)-HEPE, **E**: 15-(*S*)-HEPE, **F**: 18-(*R*-*S*)-HEPE.

**Supporting Table 1.** Sample list with running order of the LC-HRMS sequence. Cell density was determined in a Fuchs-Rosenthal counting chamber. SM: *S. maronoi*. The average cell count was 151000 and 181000 cells. mL ^-1^ for *S. marinoi* (n = 4) and evolved *S. marinoi* (n = 4), respectively.

| Sequence order | Sample name | Condition | Cell density (cells. mL ^-1^) | Normalization factor |
| --- | --- | --- | --- | --- |
| 1 | Blank1_MeOH_01 |  | | |
| 2 | Blank1_MeOH_02 |  |  |  |
| 3 | Blank2_ASW_01 |  |  |  |
| 4 | Blank2_ASW_02 |  |  |  |
| 5 | QC 01 |  |  |  |
| 6 | QC 02 |  |  |  |
| 7 | SM_S_04 | *S. marinoi* | 144500 | 0.771 |
| 8 | SM_R_04 | Evolved *S. marinoi* cycle 11 | 175100 | 0.934 |
| 9 | SM_R_03 | Evolved *S. marinoi* cycle 11 | 183000 | 0.976 |
| 10 | SM_R_02 | Evolved *S. marinoi* cycle 11 | 187500 | 1.000 |
| 11 | QC_03 |  | | |
| 12 | Blank1_MeOH_03 |  |  |  |
| 13 | SM_S_03 | *S marinoi* | 186900 | 0.997 |
| 14 | SM_R_01 | Evolved *S. marinoi* cycle 11 | 180200 | 0.961 |
| 15 | SM_S_02 | *S marinoi* | 146000 | 0.779 |
| 16 | SM_S_01 | *S marinoi* | 125600 | 0.670 |
| 17 | QC_04 |  | | |
| 18 | Blank1_MeOH_04 |  |  |  |
| 19 | Blank1_MeOH_05 |  |  |  |

**Supporting Table 2.** Suggested annotation of up-regulated metabolites in evolved cells, organized by their retention time (RT in minutes). The metabolites are elucidated by exact mass MS/MS and library comparison. Further, metabolites with proven identity by comparison with reference standards are indicated with (*). PE: phosphatidilethanolamine, PG: phosphatidilglycerol, PS: phosphatidylserine, KOT: keto-octadecatrienoic acid, MGMG: glycosylmonoacylglycerol, HDHA: hydroxydocosahexaenoic acid, HEPE: hydroxyeicosapentaenoic acid, DGDG:digalactosyl-diacylglycerol.

| **RT**  **(min)** | **Observed mass (*m/z*)** | **Mass deviation (ppm)** | **Chemical formula** | **Compound name** | **Identifier CHEBI/**  **PubChem ID** | **Diagnostic fragments**  **(*m/z*)** | **Adduct** | ***P* Value** | **Log2fold change** |
| --- | --- | --- | --- | --- | --- | --- | --- | --- | --- |
| 3.0 | 187.0975 | -0.51 | C_9_H_16_O_4_ | azelaic acid | CHEBI:132955 | 68.7260, 92.7933, 95.6425 | [M-H]^-^ | 0.020 | 1.29 |
| 4.7 | 306.0763 | -0.56 | C_10_H_17_N_3_O_6_S | glutathione | CHEBI:16856 | 60.4978, 74,0247, 99.0562, 128.0354, 143.0462, 160.0077, 254.0785 | [M-H]^-^ | 0.000015 | 2.39 |
| 5.0 | 694.3204 | -0.85 | C_29_H_50_NO_12_P | PKOHA-PS | CHEBI:168739 | 78.9591, 170.5085, 168.04337, 386.1234, 474.6695 | [M-H+HAc]^-^ | 0.014 | 3.20 |
| 5.0 | 351.2170 | -2.06 | C_20_H_32_O_5_ | prostaglandin E_2_* | CHEBI:15551 | 59.0142, 189.1283, 271.2063, 315.1979, 333.2112 | [M-H]^-^ | 0.049 | 1.26 |
| 5.3 | 532.2676 | -1.05 | C_23_H_40_NO_7_P | lysoPE (18:4) | CHEBI:170330 | 78.95906, 152.9963, 203.1805 | [M-H+HAc]^-^ | 0.00061 | 1.86 |
| 5.4 | 291.1964 | -0.65 | C_18_H_28_O_3_ | 12-KOT | CHEBI:197235 | 92.9957, 116.9956, 186.99916, 247.2080 | [M-H]^-^ | 0.0042 | 1.68 |
| 5.5 | 534.2832 | -1.03 | C_23_H_42_NO_7_P | lysoPE (18:3) | CHEBI:145279 | 78.9590, 203.4633, 249.1859 | [M-H+HAc]^-^ | 0.0023 | 1.72 |
| 5.6 | 277.1807 | -0.83 | C_17_H_26_O_3_ | 12-KHT | CHEBI:195372 | 140.9963, 233.1905 | [M-H]^-^ | 0.0000051 | 6.35 |
| 5.8 | 536.2988 | -1.14 | C_23_H_44_NO_7_P | lysoPE (18:2) | CHEBI:131744 | 78.9591, 251.2016, 476.2774 | [M-H+HAc]^-^ | 0.012 | 1.37 |
| 5.8 | 309.2069 | -0.65 | C_18_H_30_O_4_ | 13-HpOTrE | CHEBI:183350 | 73.0295, 117.0193, 179.10776, 291.1965 | [M-H]^-^ | 0.0058 | 1.47 |
| 5.9 | 560.2990 | -0.89 | C_25_H_44_NO_7_P | lysoPE (20:4) | CHEBI:72746 | 78.9591, 152.9959, 231.2114 | [M-H+HAc]^-^ | 0.00044 | 1.97 |
| 5.9 | 453.2254 | -1.14 | C_20_H_39_O_9_P | lysoPG (14:1) | CHEBI:185925 | 78..9590, 152.9960, 175.7900, 199.786, 225.1858 | [M-H]^-^ | 0.012 | 1.77 |
| 5.9 | 512.2990 | -0.93 | C_21_H_44_NO_7_P | lysoPE (16:0) | CHEBI:73004 | 79.9650, 85.0674, 227.2017, 350.0289 | [M-H+HAc]^-^ | 0.017 | 1.38 |
| 6.0 | 531.2806 | -0.97 | C_25_H_42_O_9_ | MGMG (16:3) | PubChem: 102119785 | 78.9591, 249.1860, 253.0934 | [M+FA-H]^-^ | 0.019 | 1.22 |
| 6.0 | 562.3147 | -0.79 | C_25_H_46_NO_7_P | lysoPE (20:3) | CHEBI:143227 | 78.9592, 277.2173, 502.2958 | [M-H+HAc]^-^ | 0.0041 | 1.75 |
| 6.0 | 498.2620 | -1.14 | C_25_H_42_NO_7_P | lysoPE (20:5) | CHEBI:132559 | 78.9591, 140.0119, 214.0487, 257.2273 | [M-H]^-^ | 0.010 | 1.45 |
| 6.0 | 586.3147 | -0.69 | C_27_H_46_NO_7_P | lysoPE (22:5) | CHEBI:145292 | 78.9591, 152.9959, 242.0797, 257.2274 | [M-H+HAc]^-^ | 0.014 | 1.52 |
| 6.1 | 538.3145 | -1.07 | C_23_H_46_NO_7_P | lysoPE (18:1) | CHEBI:136140 | 78.9591, 253.2173, 478.2920 | [M-H+HAc]^-^ | 0.019 | 1.34 |
| 6.3 | 588.3303 | -0.75 | C_27_H_48_NO_7_P | lysoPE (22:4) | CHEBI:136141 | 78.9592, 303.2331, 367.0465 | [M-H+HAc]^-^ | 0.025 | 1.11 |
| 6.3 | 287.1497 | -0.98 | C_12_H_20_O_4_ | traumatic acid | CHEBI:545687 | 59.0140, 90.8490, 155.1081, 201.1132, 217.1087, 227.1284 | [M-H+HAc]^-^ | 0.0086 | 1.88 |
| 6.3 | 325.2018 | -0.89 | C_18_H_30_O_5_ | 16-E1t-PhytoP | Pubchem:131839858 | 125.0976, 153.0928, 171.1028, 304.9850 | [M-H]^-^ | 0.0026 | 1.62 |
| 6.4 | 524.2778 | -0.88 | C_27_H_44_NO_7_P | lysoPE (22:6) | CHEBI:133432 | 78.9591, 214.0481, 283.2424,327.2329 | [M-H]^-^ | 0.0049 | 1.50 |
| 6.4 | 498.2257 | -1.04 | C_24_H_40_NO_9_P | lysoPS (18:4) | CHEBI:185010 | 69.0347, 78.9591, 257.1908, 301.1810 | [M-H-H_2_O]^-^ | 0.0040 | 1.06 |
| 6.4 | 564.3304 | -0.77 | C_25_H_48_NO_7_P | lysoPE (20:2) | CHEBI:145284 | 78.9591, 242.0788, 279.2328 | [M-H+HAc]^-^ | 0.0026 | 2.27 |
| 6.9 | 317.2120 | -0.85 | C_20_H_30_O_3_ | 5-HEPE* | CHEBI:140934 | 59.0140, 115.0395, 201.1646, 255.2096, 299.2041 | [M-H]^-^ | 0.014 | 1.51 |
| 7.3 | 343.2344 | -2.07 | C_22_H_32_O_3_ | 17-HDHA* | CHEBI:72637 | 59.0137, 121.0653, 201.1651, 281.2260, 325.2152 | [M-H]^-^ | 0.013 | 1.47 |
| 8.7 | 303.2328 | -0.54 | C_20_H_32_O_2_ | arachidonic acid* | CHEBI:15843 | 57.0347, 71.0138, 205.1958, 259.2431 | [M-H]^-^ | 0.018 | 1.61 |
| 9.0 | 975.5303 | -1.80 | C_51_H_78_O_15_ | DGDG (18:5/18:4) | PubChem: 52922088 | 117.2929, 253.5645, 301.2162, 384.1782, 962.5176 | [M+FA-H]^-^ | 0.000037 | 2.86 |
| 9.0 | 329.2484 | -0.78 | C_22_H_34_O_2_ | docosapentaenoic acid (DPA) | CHEBI:61204 | 71.2010, 129.6303, 251.3300, 260.1804, 285.2575 | [M-H]^-^ | 0.00036 | 5.11 |

*
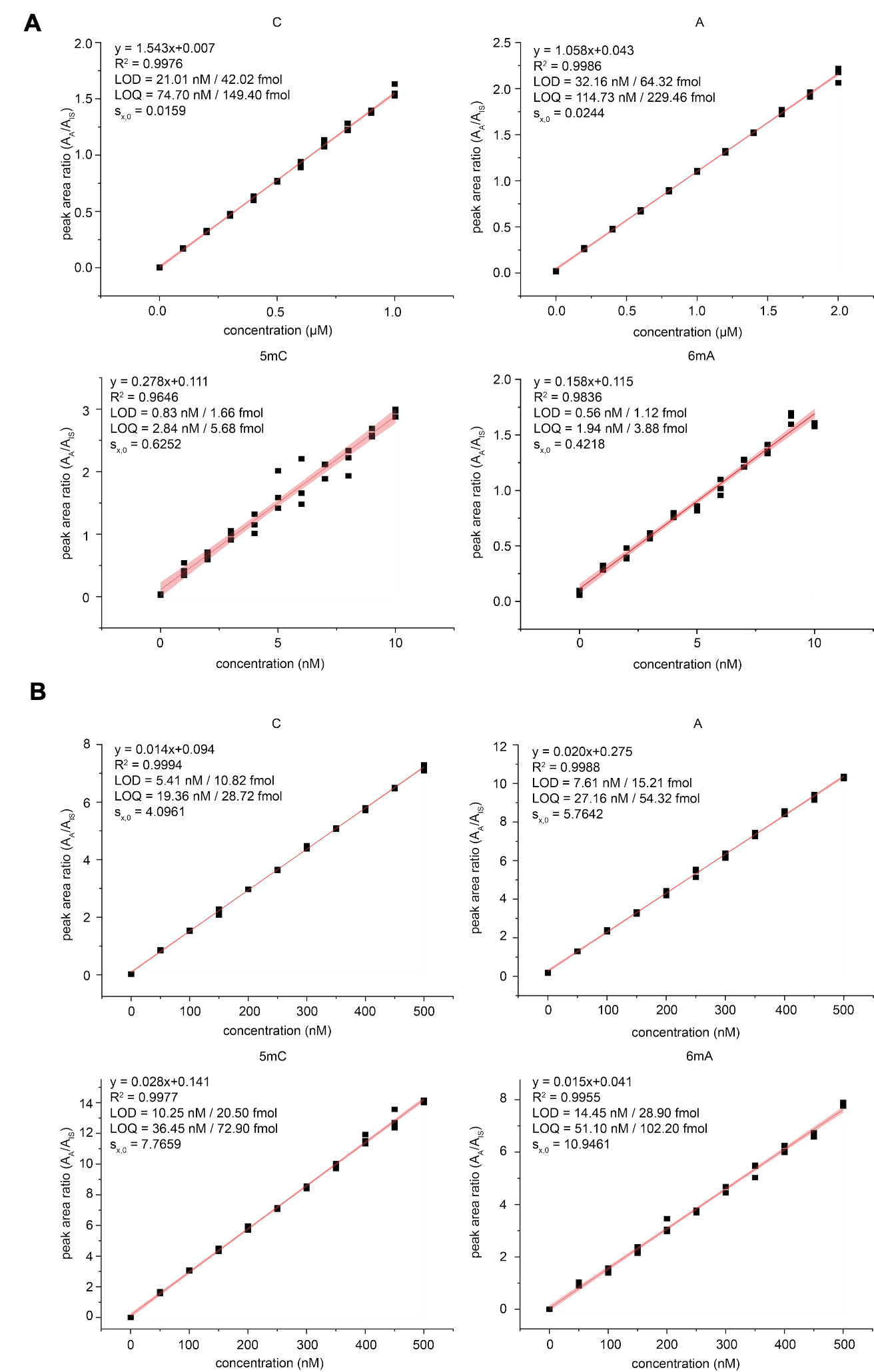
*

**Supporting Figure 10.** Calibration curves for absolute quantification of 5mC and 6mA were determined using UHPLC-HRMS Orbitrap (A: adenine, C: Cytosine, 5mC: 5-methylcytosine, 6mA: 6-methyladenine). a. for low concentrations, b for high concentrations
